# The Excitotoxin Quinolinic Acid Induces Tau Phosphorylation in Human Neurons

**DOI:** 10.1371/journal.pone.0006344

**Published:** 2009-07-22

**Authors:** Abdur Rahman, Kaka Ting, Karen M. Cullen, Nady Braidy, Bruce J. Brew, Gilles J. Guillemin

**Affiliations:** 1 Department of Family Sciences, College for Women, Kuwait University, Shuwaikh, Kuwait; 2 St Vincent's Hospital, Centre for Applied Medical Research, Department of Neuroimmunology, Darlinghurst, New South Wales, Australia; 3 Disciplines of Anatomy and Histology, School of Medical Science, The University of Sydney, New South Wales, Australia; 4 Department of Pharmacology, University of New South Wales, School of Medical Science, Sydney, New South Wales, Australia; 5 Department of Neurology, St Vincent's Hospital, Darlinghurst, New South Wales, Australia; Tufts University, United States of America

## Abstract

Some of the tryptophan catabolites produced through the kynurenine pathway (KP), and more particularly the excitotoxin quinolinic acid (QA), are likely to play a role in the pathogenesis of Alzheimer's disease (AD). We have previously shown that the KP is over activated in AD brain and that QA accumulates in amyloid plaques and within dystrophic neurons. We hypothesized that QA in pathophysiological concentrations affects tau phosphorylation. Using immunohistochemistry, we found that QA is co-localized with hyperphosphorylated tau (HPT) within cortical neurons in AD brain. We then investigated *in vitro* the effects of QA at various pathophysiological concentrations on tau phosphorylation in primary cultures of human neurons. Using western blot, we found that QA treatment increased the phosphorylation of tau at serine 199/202, threonine 231 and serine 396/404 in a dose dependent manner. Increased accumulation of phosphorylated tau was also confirmed by immunocytochemistry. This increase in tau phosphorylation was paralleled by a substantial decrease in the total protein phosphatase activity. A substantial decrease in PP2A expression and modest decrease in PP1 expression were observed in neuronal cultures treated with QA. These data clearly demonstrate that QA can induce tau phosphorylation at residues present in the PHF in the AD brain. To induce tau phosphorylation, QA appears to act through NMDA receptor activation similar to other agonists, glutamate and NMDA. The QA effect was abrogated by the NMDA receptor antagonist memantine. Using PCR arrays, we found that QA significantly induces 10 genes in human neurons all known to be associated with AD pathology. Of these 10 genes, 6 belong to pathways involved in tau phosphorylation and 4 of them in neuroprotection. Altogether these results indicate a likely role of QA in the AD pathology through promotion of tau phosphorylation. Understanding the mechanism of the neurotoxic effects of QA is essential in developing novel therapeutic strategies for AD.

## Introduction

**Figure 1:**
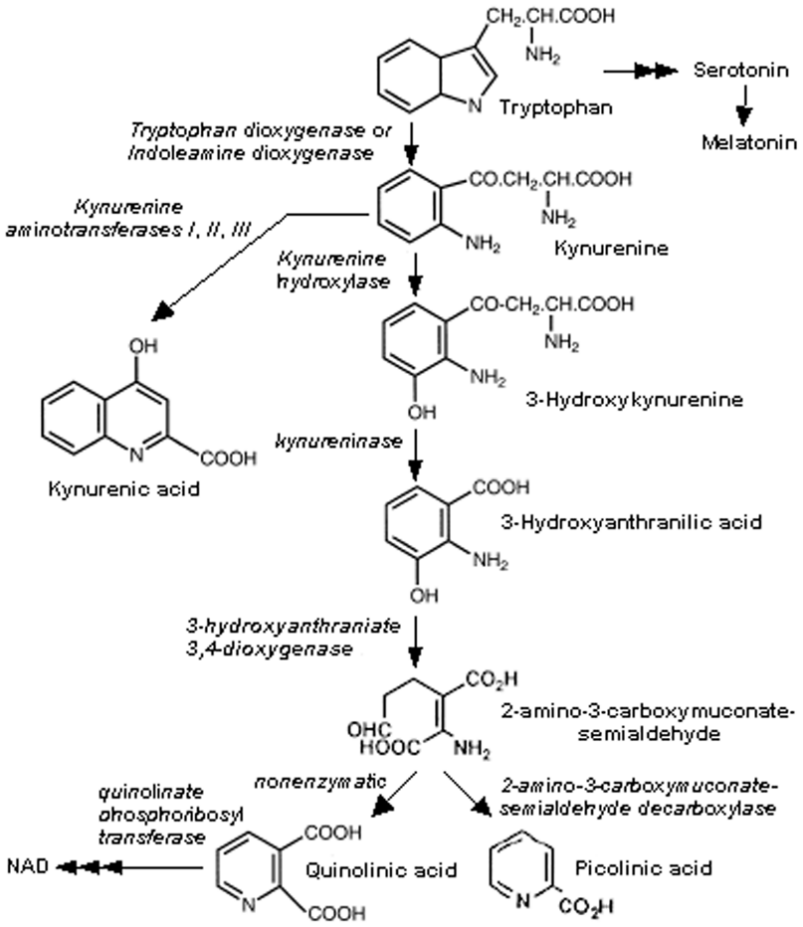
Simplified diagram of the kynurenine pathway.

The kynurenine pathway (KP) (Figure 1) is a major route of L-tryptophan catabolism, resulting in the production of nicotinamide adenine dinucleotide (NAD^+^) and other neuroactive intermediates[1]. Within the CNS, KP metabolites can have either neurotoxic or neuroprotective effects. Among them, quinolinic acid (QA) is perhaps the most important in terms of biological activity. Stone and Perkins were the first to demonstrated QA ability to selectively activate neurons expressing NMDA receptors[2]. QA neurotoxicity was then shown by Schwarcz[3]. QA leads acutely to human neuronal death and chronically to dysfunction by at least five mechanisms[4] and is known to be involved in several major neuroinflammatory diseases[4], [5], [6] including Alzheimer's disease (AD)[7].

A hallmark of AD and related tauopathies, such as frontotemporal dementia, is the formation and accumulation of neurofibrillary tangles (NFTs) within neurons. NFTs are composed of paired helical filaments (PHF)[8], [9] of which the microtubule associated protein tau in an abnormally hyperphosphorylated state, represents the major protein component[10], [11]. The phosphorylation state of tau determines its biological activity[12]. Physiologically, tau promotes the assembly and maintenance of the microtubule structures. Abnormally hyperphosphorylated tau (HPT) has less affinity for binding with microtubules. HPT also sequesters normal tau and other microtubule-associated proteins (MAP1 and MAP2) and causes disassembly of microtubules[13].

We have previously shown that the KP is activated in AD brain, leading to an increase in QA production[14]. We also found that in the hippocampus of AD patients, QA immunoreactivity is detected in cortical microglia, astrocytes and neurons. Of particular interest is the high immunoreactivity of QA in the perimeter of senile plaques and NFTs[15]. Human neurons do not produce QA but they can take it up[16], [17]. The presence of a strong QA immunoreactivity in vacuoles of hippocampal and entorhinal neurons[15] is more likely the consequence of an uptake mechanism rather than synthesis of QA. Neuronal uptake of QA is likely to be part of a scavenging process in which activated microglia and/or infiltrating macrophages release high concentration of QA[16], [17]. *In vitro*, chronic exposure of rat corticostriatal cultures to nanomolar QA concentrations leads to ultrastructural changes in neurons[18]. Moreover, both low level chronic (350 nM) and high level acute (1 µM) exposures of human primary neurons to QA are neurotoxic[19]. Indeed, chronic exposure of human neurons to QA causes significant structural changes including dendritic beading, microtubular disruption and a decrease in organelles. Altogether, the activation of the KP and the over-production of QA in AD brain, and the co-localization of QA with the histopathological lesions, particularly the QA accumulation in dystrophic neurons with the NFTs led us to hypothesize that QA is directly involved in the hyperphosphorylation of tau.

We found that QA accumulates in neurons and co-localizes with tau in cortical sections from AD patients. In addition, *in vitro* treatment of human foetal neurons with pathophysiological concentrations of QA significantly increased tau phosphorylation at multiple phosphorylation sites. Our data also show a decrease in the expression and activity of serine/threonine protein phosphatase(s), as the mechanism for tau hyperphosphorylation. We also found that QA induces at least ten neuronal genes, with some of them known to be strongly associated with Alzheimer pathology. This study provides a new insight into the mechanism of QA neurotoxicity and its important role in the pathogenesis of AD. This work has opened a new avenue for research on AD, in particular the application of a novel therapeutic target.

## Materials and Methods

### Reagents and chemicals

All neuronal cell culture media and additives were from Invitrogen (Mulgrave, VIC, Australia) unless otherwise stated. DAPI and mouse monoclonal antibodies (mAb) for β-actin and all chemicals used for Western blots, immunoprecipitation, Phosphatase (PP) activity and immunoblots (unless otherwise mentioned) were obtained from Sigma-Aldrich Chemical Co. (Sydney, NSW, Australia). IFN-γ was purchased from R&D Systems (Australia), pAb tau from DAKO, mAbs anti-tau 5 from Abcam, tau AT8 and tau AT180 from Pierce Endogen. mAb PHF-1 (developed by Prof Peter Davies, Department of Pathology, Albert Einstein, College of Medicine) and mAb AT270 (Pierce Endogen) were generously provided by Prof Jurgen Goetz (University of Sydney, NSW, Australia). Protein phosphatase antibodies PP1c and PP2Ac were kindly provided by Alistair Sim (University of Newcastle, NSW, Australia). Secondary anti-mouse IgG, anti-rabbit Alexa -conjugated antibodies (594 and 647) and streptavidin-Alexa (594 and 647) were purchased from Molecular Probes (Invitrogen, Eugene, OR, USA). Biotinylated anti-mouse and anti-rabbit antibodies were obtained from Vector Laboratories, Burlingame CA USA). All commercial antibodies were used within the concentration ranges recommended by the manufacturer.

### Ethics statement

Human foetal tissue was obtained following informed written consent. This has been approved by the Human Ethics Committees from the University of New South Wales (UNSW Ethic approval HREC 03187). Blocks of human brain tissue were obtained from the National Neural Tissue Resource Centre (NNTRC) and the Australian Brain Bank Network. This study had the approval of the Human Ethics committee of the University of Sydney (10-2005/4/8616).

### Cell cultures

Human foetal brains were obtained from 16 to 19 week old foetuses (HREC 03187). Primary cultures of human neurons were prepared as previously described[16]. Briefly, cells were plated in culture flasks coated with Matrigel (diluted 1/20 in Neurobasal medium) and maintained in Neurobasal medium supplemented with 2% (v/v) B-27 supplement, 2 mM Glutamax, 50 mM HEPES, 200 IU/ml penicillin G, 200 *µ*g/ml streptomycin sulphate and 5 mM glucose. Cultures were kept at 37°C at 5% CO_2_ in a humidified atmosphere. The medium was changed once or twice per week. Neurons were maintained for up to 10 weeks.

### Immunocytochemistry for tau

The method has been previously described[16]. Human foetal neurons were grown at a density of 5×10^6^ in Permanox chamber-slides (NUNC) for 2 weeks. Then, cells were fixed with acetone/methanol (vol/vol) for 20 min at –20°C. Cells were then rinsed 3 times with PBS and a gentle membranous permeabilization was performed by incubation with 0.025% Triton X 100 in PBS for 10 min at room temperature. After washing, cells were incubated with 5% normal goat serum (NGS) in PBS for 45 min at room temperature, rinsed twice with PBS and incubated for 1 h at 37°C with selected primary antibody Tau mAbs (Tau 5, AT8 and AT180) diluted in 5% NGS. Cells were then washed with 5% NGS solution and incubated for 1 h at 37°C with the appropriate labelled secondary antibodies (goat anti-mouse IgG or goat anti-rabbit coupled with Alexa 594). Nuclear staining was performed using DAPI at 1 µg/ml for 5 min at room temperature. After several washings with PBS at 37°C, the cover slips were quickly mounted on glass slides with Fluoromount-G, and examined using an Olympus BX60 fluorescence microscope associated with a digital SensiCam. The following three controls were performed for each labelling experiment: 1) isotypic antibody controls for mAbs and serum control for pAbs, 2) incubation with only the secondary labelled antibodies, 3) estimation of auto-fluorescence of unlabelled cells.

### Quinolinic acid immunohistochemistry in AD brain tissue

Increased QA expression was confirmed in AD tissue. Brain tissue was acquired through the NSW and Victorian Brain Bank Networks supported by the National Health and Medical Research Council. The study has the approval of the Human Ethics Committees at the University of Sydney. Two age matched controls and 8 AD cases with a range of neuropathological severity were examined. Blocks of tissue were taken from 3–5 mm formalin-fixed coronal slices of the medial temporal lobe, anterior cingulate and superior frontal cortex. On a CO_2_ Leica microtome, blocks were sectioned at 45 µm into 0.1 M Tris buffer (pH 7.4) containing 0.01% azide. Serial sections were labelled for QA (mAb, developed by Chemicon for our group) and tau (AT8, AT180, Pierce Endogen). Free floating sections were washed three times for 15 minutes each in 50% ethanol then placed in blocking buffer (0.1 M Tris, 1% bovine serum albumin, 0.01% azide, 0.01% TritonX) for 20 minutes, then incubated in 1° antibody for 24 hrs at 4°C. Sections were washed three times for 15 minutes each in 0.1 M Tris and incubated in species-specific biotinylated antibody for 1 hr, washed and placed in streptavidin-Alexa 647 or 594 (Molecular Probes, Invitrogen) diluted 1∶100 in 0.1 M Tris with 0.01% TritonX for 1 hr. For double-immunostaining, the sections were washed for one hour, incubated in blocking buffer, then transferred to the second primary antibody solution. For viewing, sections were mounted on gelatinized glass slides, cover-slipped in gelatine/glycerol and examined with a Zeiss Axioplan microscope. Optical sections were taken through the sections at 2 µm intervals and reconstructed using Zeiss Axiovision software and ImageJ (NIH; Rasband, W.S., ImageJ, U. S. National Institutes of Health, Bethesda, Maryland, USA, http://rsb.info.nih.gov/ij/, 1997–2008).

### Preparation of neuronal lysates for Western blots

For Western blots, cells were cultured in Matrigel coated 12-well plates at a density of 1×10^6^ cells per well in supplemented Neurobasal medium supplemented (as described above). Neurons were incubated with various concentrations of QA (ranging from 50 to 1200 nM) for 72 hours. Control cultures were kept in the medium alone. Okadaic acid (100 nM) was used as positive control for tau phosphorylation. In a separate experiment, neurons were cultured with 500 nM each of NMDA receptor agonists (QA, glutamate, NMDA) and antagonists (memantine, AP-5 and MK-801) for 72 hrs. Cells were lysed in 500 µl of RIFA buffer, (50 mM Tris, pH 7.4, 150 mM NaCl, 1% NP-40, 5 mM EDTA, 0.5% sod. deoxycholate, 0.1% SDS, 50 nM NaF, 1 mM sodium vanadate, 2 µg/ml aprotinin, 2 µg/ml pepstatin and 5 µg/ml leupeptin). The cell lysate was centrifuged at 16000 g for 10 minutes. Protein concentration in the supernatant was determined by the modified Lowry method and stored at −20°C until used.

### SDS-PAGE and immunoblot analyses

20 µg lysate protein was resolved on 10% SDS-PAGE (NuPAGE 10% from Invitrogen, Carlsbad CA USA) in sample buffer. Protein was transferred onto PVDF membranes; the membranes were blocked with 5% milk in TBS for one hour and incubated with primary antibody overnight at 4°C. The primary antibodies used are described in Table 1. After incubation, the membranes were rinsed several times, washed with TBS-tween three times (10 minutes each), and then two times with TBS only. The membranes were then incubated with the secondary antibody for 4 hours at RT, washed as before and developed either with ECL or Chemidox, (Biorad, Hercules, CA, USA) according to the manufacturer instructions. Blots were quantified using the Quantity One 4.6.1 software from Biorad and ImageJ (NIH; Rasband, W.S., ImageJ, U. S. National Institutes of Health, Bethesda, Maryland, USA, http://rsb.info.nih.gov/ij/, 1997–2008).

**Table 1:** List of primary antibodies used.

Antibody	Type	Antigen	Source/Reference
Tau	polyclonal	Total tau	DAKO
AT8	monoclonal	pS199/202	Innogenetics
AT180	monoclonal	pT231	Innogenetics
AT270	monoclonal	pT181	Innogenetics/Gotz
PHF-1	monoclonal	against pS396/404	Peter Davies
Anti-β-actin	monoclonal	β-actin	Sigma
Anti-PP1 gamma 1	polyclonal	PP1c	Sigma
Anti-PP2A (clone 1D6)	monoclonal	PP2Ac	Upstate
Anti-PP5	polyclonal	PP5	Sigma
Anti-calcineurin (clone CNA1)	monoclonal	PP2B catalytic subunit	Sigma
Anti-Quinolinic acid	monoclonal	QUIN	Chemicon

### Phosphatase activity assay

For phosphatase activity, cells were cultured as described above and lysed in the phosphatase activity buffer (50 mM Tris, pH 7.5, 150 mM NaCl, 1% NP-40, 1 mM PMSF, 1 mM benzamidine, 10 mM beta-mercaptoethanol (βME), 2 µg/ml aprotinin, 2 µg/ml pepstatin and 5 µg/ml leupeptin. Total phosphatase activity was determined using pNPP (10 mM) as a substrate in 50 mM Tris-HCl, pH 7.4, 0.1 mg/ml BSA, 3 mM CaCl_2_, 1 µM calmodulin and 3 mM MnCl_2_. The reaction mixture was incubated at 30°C for 30 min and the absorbance measured at 405 nm. PP2A activity was indirectly measured by using 10 nM OA in the phosphatase assay buffer and calculating the OA-inhibited activity.

### Immunoprecipitation of PP2A

PP2A was immunoprecipitated according the protocol provided by Upstate, New York. Briefly, 4 µg of anti-PP2A (Clone 1D6) (Upstate, New York) was incubated with 100 µl Protein A Sepharose beads (Amersham/Pharmacia) for 4 hours at 4°C on a rotor. The beads were washed with 750 µl TBS 3 times and 2 times with the phosphates lysis buffer. The beads with absorbed anti-PP2A were incubated with 400 µg lysate protein in a total volume of 500 µl overnight at 4°C. Unbound proteins were separated, and the beads were washed (by a brief pulse in table-top centrifuge) with 750 µl of TBS 3 times. For phosphatase activity, 20 µl beads were pelleted and resuspended in 200 µl of phosphatase assay buffer containing 50 mM Tris-HCl, pH 7.4, 3 mM MnCl_2_ and 10 mM pNPP. The reaction mixture was incubated at 30°C for 30 min with occasional mixing. At the end of incubation, the beads were centrifuged and the clear supernatant was collected into the wells of a 96-well ELISA plate and the absorbance measured at 405 nm. To measure the amount of immunoprecipated PP2A, 20 µl of beads were boiled in 4× sample buffer and subjected to SDS-PAGE and Western blots as described earlier.

### PCR array

RT^2^ Profiler™ PCR Array kit were obtained from SuperArray Bioscience Corporation (Frederick, MD, USA). The Human Signal Transduction PathwayFinder™ RT^2^ *Profiler*™ PCR Array profiles the expression of 84 key genes representative of 18 different signal transduction pathways. Using real-time PCR, we analysed the expression of a focused panel of genes related to any of the 18 signal transduction pathways involved in the response of human primary neurons to QA 150 nM for 24 h.

## Results

### Quinolinic acid is found in neurons and microglia in AD cortex

**Figure 2:**
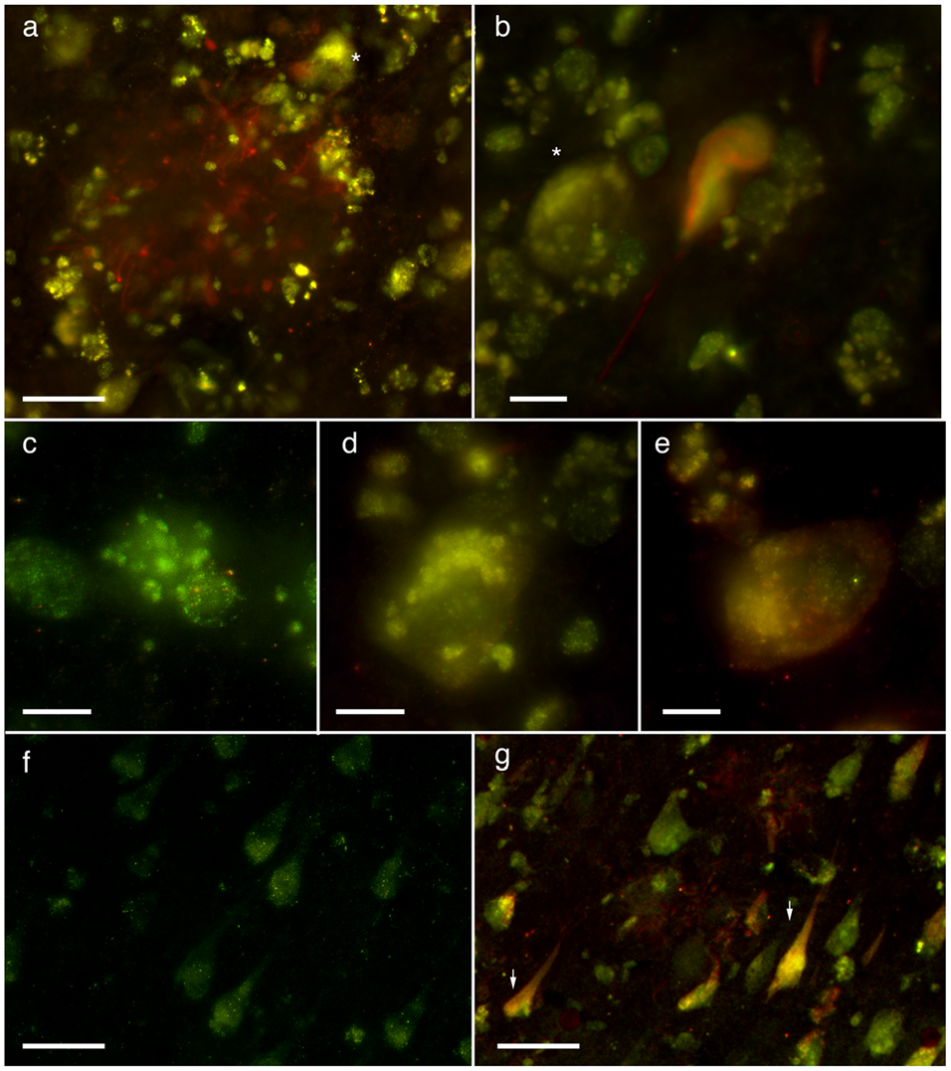
Immunodetection of tau and QA in AD sections. QA (red; Alexa 647) and tau (AT8; green; Alexa 594) immunolabelling in control and AD cingulate cortex (a–e) and hippocampus (f–g). a) Neuritic plaque in the cingulate cortex of a late-stage AD showing the AT8-positive neurites surrounding by QA-positive cells. The majority of this labelling can be seen in vesicle-like structures within microglial cells, however QA+ neurons in the plaque vicinity are also present (asterix). b) Co-localisation of an intracellular neurofibrillary tangle and QA. This tangle containing neuron is contacted by QA+ non-neuronal cells. See also supplementary figure S3 for additional views through this neuron. c) QA-positive vesicles within a microglia and d) neuron. e) Punctate tau-positivity in a neuron also positive for QA. f) 73-year old neurologically normal control hippocampal CA1 pyramidal cells with very faint immunopositivity for QA. This case had small numbers of entrohinal tangles, but otherwise no other AD-type lesions were detected. In non-AD cases, QA+ neurons rarely occurred outside the hippocampal-entorhinal sector. g) Hippocampus of a mild AD case (Braak stage IV) with QA co-localisation with AT8+ intraneuronal tangles. Scale bars 10 µm a–e; 50 µm f–g.

In order to validate the relevance of QA in the mechanism of tau phosphorylation in the human disease, AD and control tissue were labelled for QA and tau (AT8, AT180). We confirmed that QA is found within cortical neurons in AD and, to a substantially lesser degree, in cortical neurons of elderly controls (**Figure 2**). QA is present intrasomally in punctate structures resembling vesicles and co-localizes with tau-positive fibrillary structures (see also supplementary Figure S3). QA is also seen throughout the parenchyma and enriched within plaques in vesicular structures within microglial processes.

### Quinolinic acid treatment induces tau phosphorylation in cultured human foetal neurons

**Figure 3:**
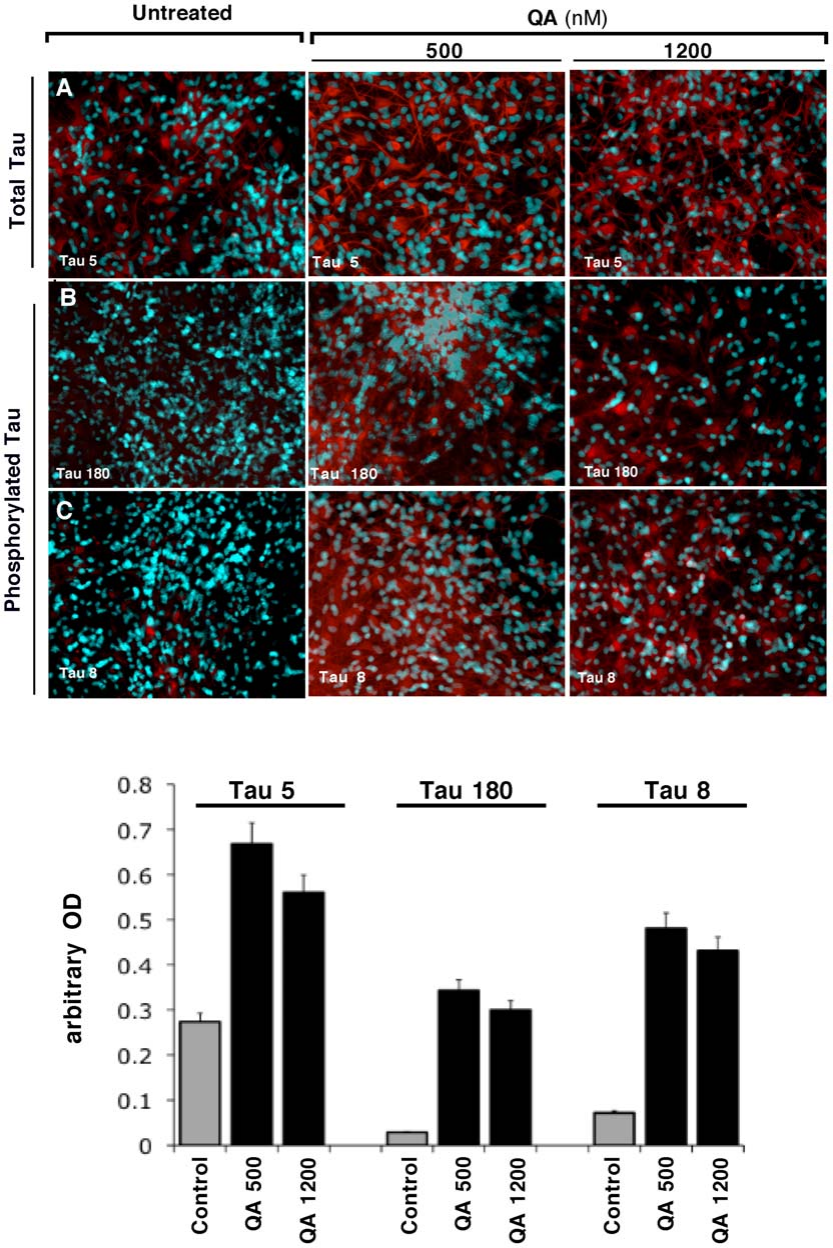
Immunodetection of Tau Phosphorylation in primary cultures of human neurons. Primary cultures of human neurons were immunostained for tau phosphorylation with three different antibodies: Line A, staining of total tau using mAb Tau 5 (Abcam); line B, staining of phosphorylated tau using mAb Tau 180 (Innogenetics); and line C, staining of phosphorylated tau using mAb Tau 8 (Innogenetics). The left column corresponds to untreated neurons, the middle column to QA 500 nM treated cells and the right column to QA 1200 nM treated cells. The histograms represent the semi-quantification of the immunostaining intensity. Intensity has been quantified using ImageJ 10.2. This experiment has been performed in triplicate with primary cultures of neurons prepared from three different brains. Three individual microscopic fields were analysed for each treatment and the SEM for the data was determined to be <5%.

QA 500 and 1200 nM significantly increased not only the level of total tau (**Figure 3A**), but also tau phosphorylation at the Tau-8 and Tau-180 epitopes (**Figure 3B and C**, respectively), as shown by the increase in the staining intensity. Cultures treated with 1200 nM QA had less DAPI-stained neurons and a lower intensity of red staining, compared to control culture and cells treated with 500 nM QA, suggesting that QA 1200 nM is neurotoxic.

**Figure 4:**
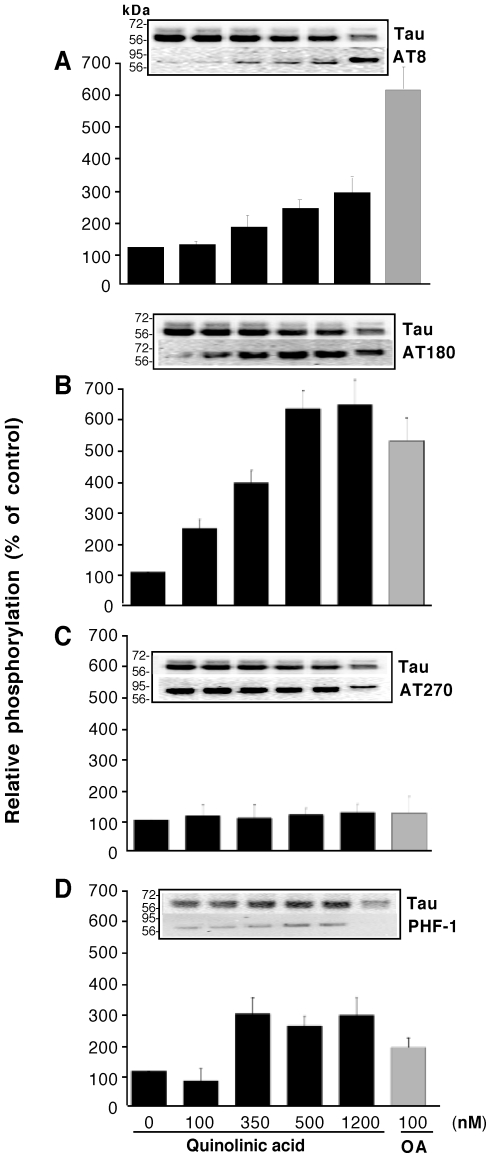
QA induces tau phosphorylation at multiple serine and threonine residues: Primary human foetal neurons were cultured at a density of 1×10^6^ cells per well in a 12-well plate in the absence or presence various concentrations of QA. 100 nM Okadaic acid was used as positive control for tau phosphorylation. After 72 hours, cells were lysed in RIFA buffer. The lysate protein resolved in 10% SDS-PAGE and Western blot performed with the antibodies indicated. Antibodies tested are: A, AT8 (1∶1000 dilution); B, AT180 (1∶1000); C, AT270 (1∶2000); D, PHF-1 (1∶1000). Signal for total tau was determined with the antibody Tau at 1∶1000 dilution. Blots were quantified using the Quantity-One software (Biorad). Signal for phospho tau epitopes was normalized by the signal for total tau and the data expressed as percent of the normalized signal in control cultures. Data presented are the mean (±SD) of five different experiments with neurons from five different brains. Each graph is accompanied with a representative blot in which lane 1 is control; lane 2, 100 nM QA; lane 3, 350 nM QA; lane 4, 500 nM QA; lane 5, 1200 nM QA; lane 6, 100 nM Okadaic acid.

**Figure 5:**
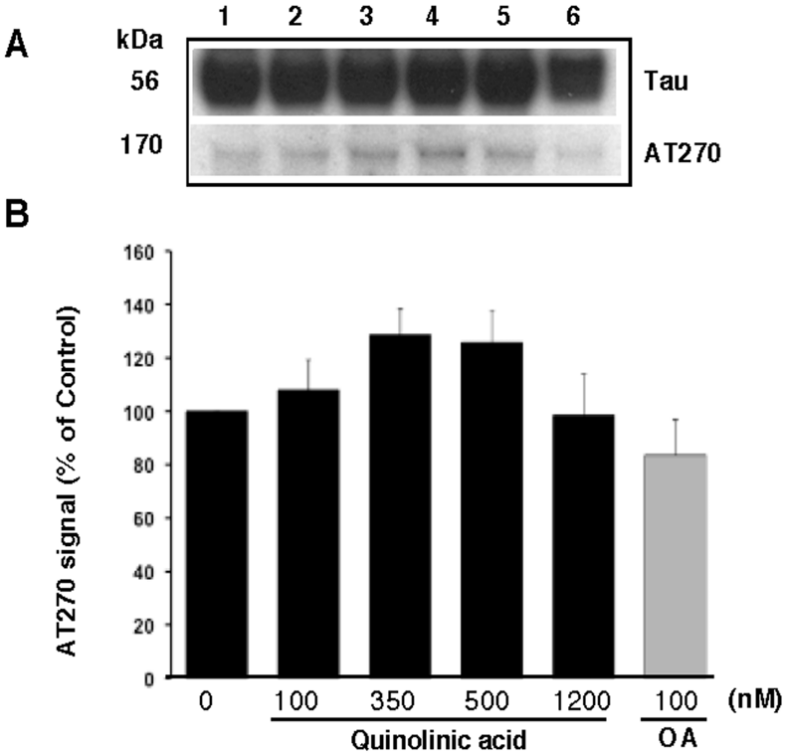
QA treatment induced the aggregation of tau: A: Representative blot showing the presence of a high molecular weight (∼165 kDa) band recognized by the PHF-specific antibody AT270 in response to QA treatment. Treatments are the same as shown in Figure 4. B: Quantitative estimation of the high molecular weight tau band normalized for total tau. The data presented are the average (±SD) of four experiments from four different brains and are expressed as the ratio of signal for AT270 to that of signal for total tau.

To further elucidate the effect of QA on tau phosphorylation, human foetal neuronal cultures were treated with four different concentrations of QA for 72 hours and Western blot analyses were performed using four different phosphorylation specific antibodies. Details of the experiment are given in the Materials and Methods and in the figure legend. The results are shown in Figure 4. Okadaic acid (100 nM) was used as positive control. At this concentration okadaic acid increased tau phosphorylation with all the four antibodies studied. Phosphorylation of threonine 181 (AT270) was increased by 20%, serine 199/202 (AT8) by 450%, threonine 231(AT180) by 400% and serine 396/404 (PHF-1) by 70% over the control levels by OA. QA increased phosphorylation of serine 199/202 and threonine 231 in a dose dependent manner over the basal level of phosphorylation in the control cultures (Figure 4). The effect on threonine 181 and serine 396/404 was not dose dependent. Phosphorylation of threonine 181 was slightly (20%) increased by the relatively higher concentrations of QA used, whereas, phosphorylation of serine 396/404 was increased substantially by QA at concentrations of 350 nM or higher. Control cultures showed a substantially higher level of phosphorylation of T181, compared to the other sites tested (**Figure 4**), and both QA and OA resulted in a minimal increase in the phosphorylation of this site over the control level. An interesting observation was the presence of a higher molecular weight band (∼165 kDa) detected with the antibody AT270 (pT181), which may represent aggregated tau (**Figure 5**).

### Quinolinic acid decreases the expression and activity of serine/threonine protein phosphatases in cultured human foetal neurons

**Figure 6:**
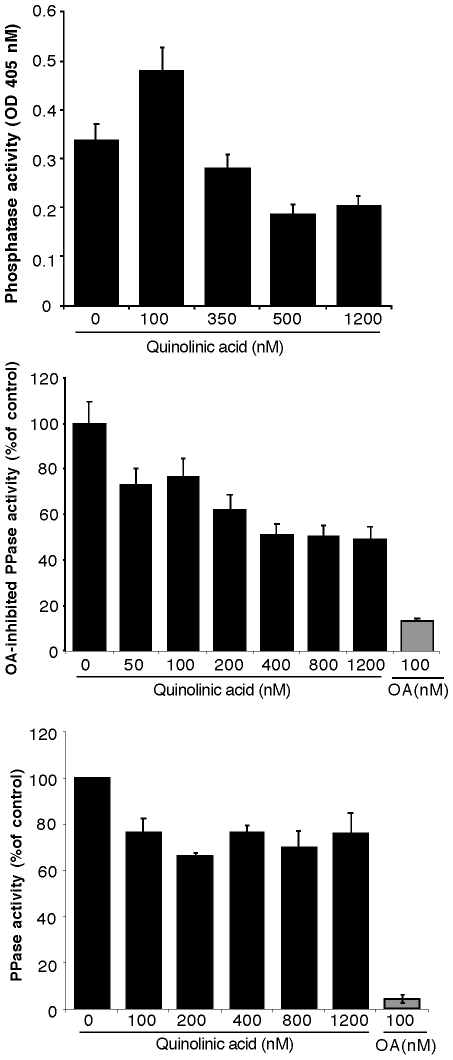
QA treatment reduces total phosphatase and PP2A activity in cultured neurons: Neuronal cultures were treated with the concentrations of QA shown, for 72 hours. Cells were lysed in the phosphatase activity buffer (50 mM Tris, pH 7.5, 150 mM NaCl, 1% NP-40, 1 mM PMSF, 1 mM Benzamidine, 10 mM β-mercaptoethanol (βME), 2 µg/ml aprotinin, 2 µg/ml pepstatin and5 µg/ml leupeptin. A: Total phosphatase activity was determined using pNPP (10 mM) as a substrate in 50 mM Tris-HCl, pH 7.4, 0.1 mg/ml BSA, 3 mM CaCl_2_, 1 µM calmodulin and 3 mM MnCl_2_. The reaction mixture was incubated at 30°C for 30 min and the absorbance measured at 405 nm. Data shown are the average (±SD) of three experiments. B: OA-inhibited (PP2A) in the lysate of neurons treated with QA. Total phosphatase activity towards pNPP was determined as above in the presence or absence of 10 nM OA. The difference in the activity lost due the addition of 10 nM OA was calculated as PP2A activity. C: Activity of immunoprecipitated PP2A towards pNPP. PP2A was immunoprecipitated from 400 µg lysate protein with anti-PP2A (clone 1D6). 20 µl of bead were resuspended in 200 µl of phosphatase activity buffer containing 50 mM Tris-HCl, pH 7.4, 3 mM MnCl_2_ and 10 mM pNPP. The reaction mixture was incubated at 30°C for 30 min with occasional mixing. Beads were removed and the OD of the clear supernatant was measured at 405 nm. Data from three separate experiments are shown as mean (±SD).

**Figure 7:**
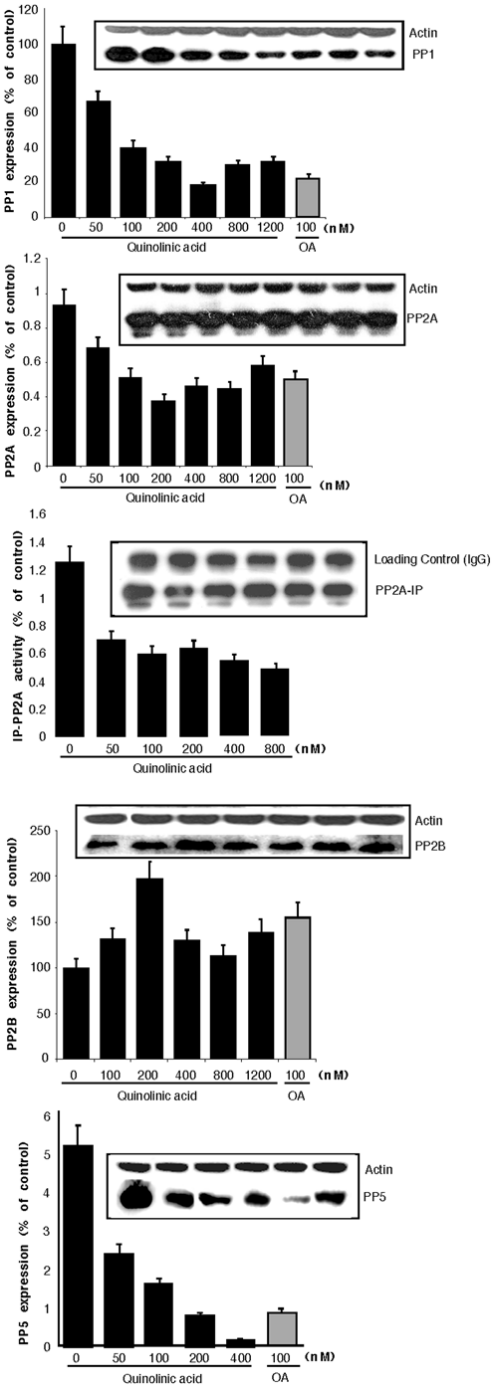
QA treatment decreases the expression of PP1, PP2 and PP5 but increases the expression of PP2B: 20 µg lysate protein (or 20 µl PP2A-IP beads for Figure 7C) was resolved on 10% SDS-PAGE, protein transferred on to PVDF membrane and probed with respective antibodies for each phosphatase. For loading control, the same membranes were stripped and re-probed with anti-actin antibody. For PP2A-IP the band of IgG heavy chain was used as loading control. Graph are mean (±SD) of three experiments (from three separate brains) and are accompanied by a representative blots. Each bar of the quantification graph represents the corresponding band above for each phosphatase.

In order to investigate if the increased phosphorylation of tau was due to a decrease in the phosphatase activity of neurons, we investigated the effect of QA on phosphatase activity using pNPP as a substrate. We observed an apparent increase in total phosphatase activity at 100 nm QA, but a dose dependent decrease thereafter at higher concentrations used (**Figure 6A**). Since PP2A is the major phosphatase activity, we indirectly measured PP2A activity in the presence of 10 nM OA, and calculated the OA-inhibited (PP2A) activity. QA inhibited OA-inhibited phosphatase activity in a dose dependent manner (**Figure 6B**). To confirm these results, we immunoprecipated PP2A from the lysate onto protein A agarose beads and measured the phosphatase activity of immunoprecipitated PP2A towards pNPP. All concentrations of QA tested decreased phosphatase activity by approximately 30%. The decrease was, however, not dose dependent (**Figure 6C**). To confirm these results, and to investigate if this apparent change in phosphatase activity was due to a change in protein expression, we measured the expression levels of PP1, PP2A, PP2B and PP5 by Western blot analyses in the lysate of neurons treated with various concentrations of QA. The results are shown in **Figure 7**. As shown in **Figure 7A**, QA treatment decreased the expression of PP1 in a dose dependent manner. QA at a relatively lower concentration of 400 nM decreased PP1 expression to the same extent as achieved by 100 nM OA. A similar dose-dependent decrease in the expression of PP2A (**Figure 7B and 7C**) and PP5 (**Figure 7E**) was also observed with QA acid treatment. PP2B expression on the other hand was increased by all concentrations of QA tested (**Figure 7D**). Maximum increase (∼200% of control) was observed with 200 nM QA. OA treatment also increased PP2B expression.

These results strongly support the hypothesis that the excitotoxin QA induces tau phosphorylation by inhibiting the activity and/or expression of multiple serine/threonine protein phosphatases (PP1, PP2A and PP5). Our data also suggest that the apparent observed increase in total phosphatase activity in the neuronal lysate treated with relatively lower concentrations of QA (up to 200 nM) may be due to an increase in PP2B expression and/or activity.

### Quinolinic Acid-induced tau phosphorylation in cultured foetal neurons involves the activation of NMDA receptor

**Figure 8:**
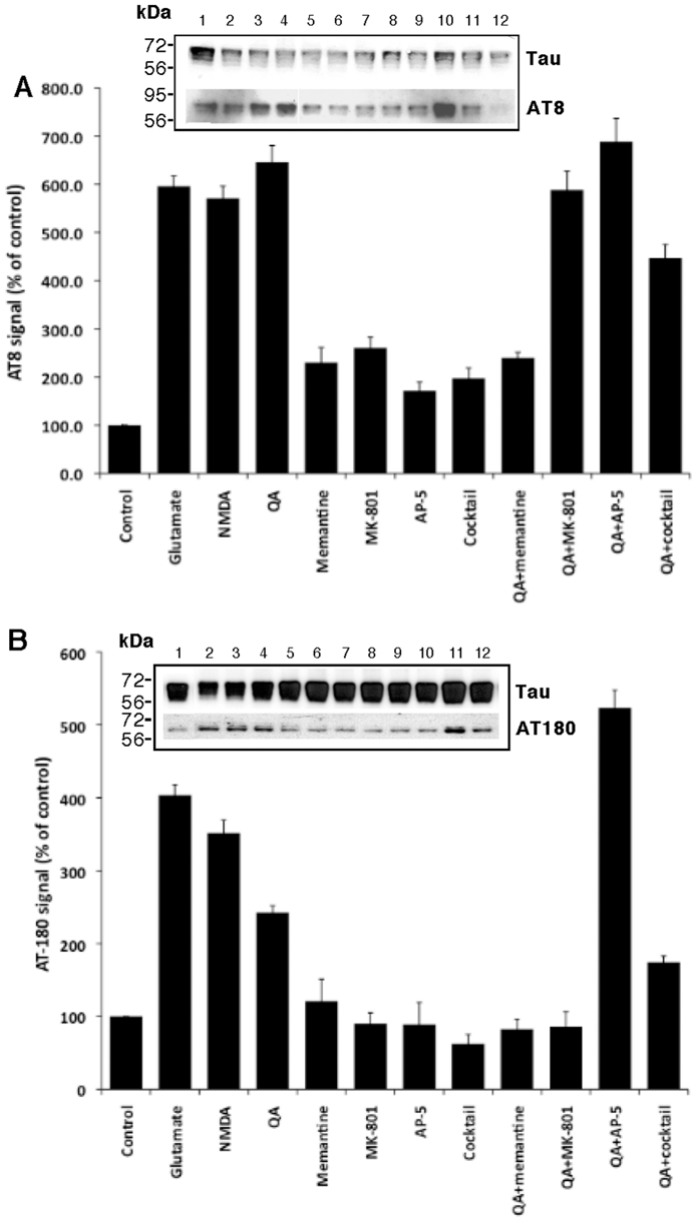
QA-induced tau phosphorylation occurs through the activation of NMDA receptor, and memantine inhibits QA-induced tau phosphorylation: Primary cultures of human neurons were treated with 500 nM each of three NMDA receptor agonists (QA, glutamate and NMDA) and three antagonists (memantine, MK-801 and AP-5). One well (termed as cocktail) was treated with a combination of all the three antagonists (500 nM each). Control wells were cultured in medium alone. Cells were cultured for 24 hours and were then lysed in RIFA buffer. 20 µg of protein lysate was resolved on 10% SDS PAGE, transferred onto PVDF membrane and probed with phosphor-specific antibodies AT8 and AT180 (1∶100 dilution). Antibody Tau (DAKO, 1∶10000) was used for total tau. Quantization data is presented as ratios of AT8 and AT-180 signal to total tau signal and is the average (±SD) of three experiments. A representative blot is for each graph is shown in the insert.

Since QA is an NMDA receptor agonist, we investigated the effect of other NMDA receptor agonists (glutamate and NMDA) on tau phosphorylation and tested whether the NMDA receptor antagonists can abrogate QA-induced tau phosphorylation. The results are shown in **Figure 8**. NMDA receptor agonists, glutamate and NMDA at equimolar concentrations (500 nM) increased tau phosphorylation at serine 199/202 (AT8) and threonine 231 (AT-180), similar to QA. Phosphorylation of tau at the AT8 site by QA was inhibited by memantine (500 nM) but not by AP-5 or MK-801. The combination of all the three antagonists (cocktail) inhibited tau phosphorylation to some degree but not to the extent of inhibition by memantine alone. Phosphorylation at the AT-180 site was inhibited by memantine and MK-801 but not by AP-5. The antagonist cocktail was less effective in inhibiting phosphorylation of threonine 231 than memantine or MK-801 alone. These results suggest that QA-induced tau phosphorylation involve NMDA receptor activation and inhibiting the NMDA receptors by an antagonist inhibits the effect of QA on tau phosphorylation.

### Quinolinic Acid treatment induces the expression of genes that are involved in AD pathology and tau phosphorylation

**Figure 9:**
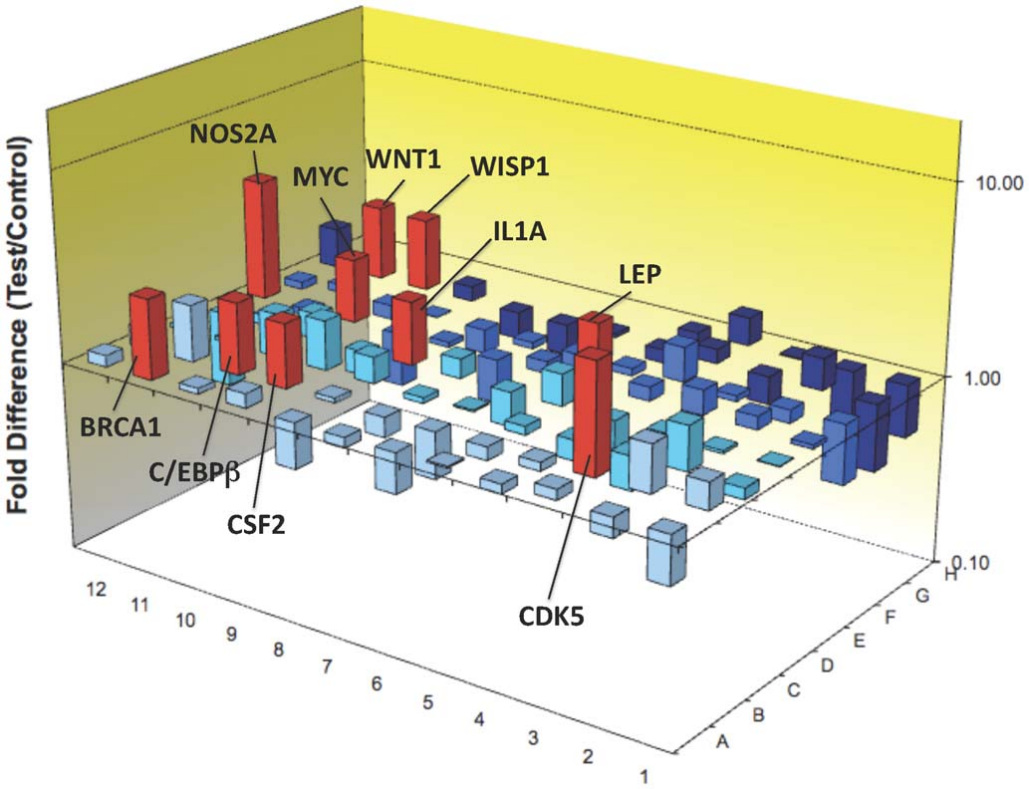
Determination of the genes induced by QA in primary cultures of human neurons using PCR arrays: The 3D histogram shows 84 genes tested in the *Pathfinder kit*®. Only 10 genes (in red) were significantly increased (expression>2x the control). No genes were significantly decreased.

We investigated the expression of genes related to several signalling pathways in neuronal cultures treated with QA for 24 hours. Of the 84 genes tested, QA induced the expression of 10 genes (**Figure 9** and Table 2) by more than two-fold over the control cultures. All these 10 genes are linked with AD pathology and 6 are known to be involved in tau phosphorylation. Details of the over-expressed genes and their reported roles in AD pathology, tau phosphorylation and neuroprotection are given in Table 2.

**Table 2:** 

Genes	Increase	Genebank	Functions	Link with AD’s pathology
BRCA1 (Breast cancer 1)	2.64	NM_007294	Cell cycle control. BRCA1 is a regulator of cell cycle	BRCA1 is found strongly expressed in all Alzheimer’s brain specifically in neurofibrillary tangles. BRCA1 is also linked with prevalence for Alzheimer’s disease[52].
MYC (c-myc myelocytomatosis viral oncogene)	2.16	NM_002467	Multifunctional nuclear phosphoprotein involved in cell cycle progression, apoptosis and cellular transformation.	Strong c-Myc immunoreactivity is associated with dystrophic neurites and neurons with NFT. Correlation between increased Tau phosphorylation and c-myc over expression in rat primary neurons[53], [54].
WNT1 (Wingless-type MMTV integration site family 1)	2.45	NM_005430	Implicated in oncogenesis, migration, adhesion and in several developmental processes,	WNT1 interferes with PP2A activity leading to tau protein hyperphosphorylation. Wnt signalling pathway links both amyloid plaques biogenesis and neurofibrillary changes observed in AD brains[55], [56], [57].
WISP1(WNT1 inducible signalling pathway protein 1)	2.39	NM_003882	Involved in developmental processes and cell proliferation	WISP1 attenuates p53-mediated apoptosis through activation of the Akt kinase. It is neuroprotective as WISP1 phosphorylates GSK-3β and maintains β-catenin to enhance neuronal survival and suppress microglial activation in AD pathogenesis[56], [57].
CDK5 (Cyclin-dependent kinase 5)	3.71	NM_014207	Cdk5 is an atypical cyclin-dependent kinase localized in the brain	CDK5 is involved in the regulation of tau phosphorylation[58].
IL1A (Interleukin 1 α)	2.14	NM_000575	Pleiotropic cytokine involved in various immune responses, inflammatory processes, and hematopoiesis.	Polymorphism of these genes is associated with Alzheimer's disease. IL1-α induces tau phosphorylation in human astrocytes[59].
NOS2A (Nitric oxide synthase 2A)	4.29	NM_000625	Commonly known as iNOS, it is inducible by LPS and inflammatory cytokines.	NOS2A/iNOS protein level is significantly increased in AD patients leading to oxidative stress. NO triggers tau hyperphosphorylation in hippocampal neurons. iNOS immunoreactive profiles were detected associated with senile plaques and extracellular neurofibrillary tangles[60], [61].
C/EBPB (CCAAT/ enhancer binding proteinβ)	2.38	NM_005194	The C/EBP is implicated in cellular injury and regeneration in neuron.	C/EBP has been reported to be up regulated in AD. Activation of NMDA receptors leads to an increase of C/EBPβ expression, which is associated with a neuroprotective anti-oxidative response and expression of regeneration-associated genes in neuron[62], [63].
CSF2 (Colony stimulating factor 2 or GM-CSF)	2.17	NM_000758	Cytokine stimulating microglial cell growth and exerting inflammatory properties.	Significantly increased CSF levels of GM-CSF in AD. GM-CSF is associated with neuroprotection and neuroplasticity[64], [65].
LEP (Leptin)	2.14	NM_000230	Linked to severe and morbid obesity. In the brain, it’s linked to synaptic plasticity.	Leptin dysregulation is associated with enhanced susceptibility of neurons to damage and it has been found to be neuroprotective in neurodegenerative brain regions[66].

## Discussion

We previously found that QA accumulates in AD brain and interestingly was seen in dystrophic hippocampal neurons [15]. We confirm this here and show co-localization with HPT in cortical and hippocampal neurons. QA is also seen in non-neuronal cells (microglia), which is likely to be the source for neuronal uptake. The present study was designed to assess the potential effect of QA on tau phosphorylation. In accord with our hypothesis, we demonstrated that QA increases tau phosphorylation and so is likely to be a critical factor involved in NFTs formation and subsequent neuronal death. Ultimately, QA accumulation could lead to a worsening of AD neuropathogenesis.

Tau is a phosphoprotein that can be phosphorylated at multiple serine and threonine residues. Hyperphosphorylated tau is assembled into paired helical filaments (PHF) leading to the formation of NFTs[11]. At least thirty serine/threonine phosphorylation sites have been identified in abnormally hyperphosphorylated tau from AD brain[20]. Mechanisms of tau hyperphosphorylation are still not fully understood. Tau hyperphosphorylation results in the disruption and disassembly of the microtubules and subsequently in neuronal death and memory loss[21]. The neuronal disruption of microtubules by hyperphosphorylated tau, which accumulates as PHF in NFTs, is one of the key features of AD[8], [22].

Several neuroinflammatory mediators are able to activate the KP leading to the production of QA by activated microglia and infiltrating macrophages[4]. Microglial activation is known to occur early in transgenic models of tauopathies and immuno-suppression attenuates tau pathology[23], providing a potential corroborating link between microglial products and tangle formation. Interestingly, other excitotoxins such as kainic acid are also able to increase formation of PHF protein[24]. QA is an NMDA receptor agonist and represents a potent endogenous excitotoxin. Increases in QA concentration are known to be associated with several neurodegenerative diseases[4], [7], [25]. We previously reported the presence of QA in close proximity to NFTs and we also observed that QA accumulates in vacuoles within the cytoplasm of dystrophic neurons. We showed here that QA and NFTs are often co-localized in the brain of AD patients[15], [26]. We found that *in vitro* human primary neurons can take up exogenous QA (see supplementary Figure S1) but can only catabolize a limited amount[16]. This is likely to be due to a rapid saturation of the catabolising enzyme for QA, quinolinate phosphoribosyltransferase (QPRTase) (EC 2.4.2.19) (Figure 1) (see supplementary Figure S2). We demonstrated that neuronal QPRTase activity begins to be saturated at QA concentrations≥300 nM. Neurons can take up and catabolise QA to produce more NAD^+^ [27], which would provide more energy to the cell and improve DNA repair[28]. The excessive accumulation of QA is likely to induce a cytotoxic cascade within neurons[15], [26].

Taken together these observations led us to investigate whether elevated concentrations of QA may contribute to AD neuropathology by leading to tau hyperphosphorylation. We found that *in vitro* QA treatment of human primary foetal neurons led to a substantial increase of tau phosphorylation at multiple positions. Sites S199/202, T231 and S396/404 are more sensitive to phosphorylation induced by QA treatment. T181 showed a higher degree of phosphorylation in the control cultures and phosphorylation at this site was not substantially increased by QA treatment. However, QA treatment increased the appearance of a high molecular band (∼165 kDa) detected with antibody AT270, which recognizes pT181. This suggests that QA-induced phosphorylation at this site may increase the aggregation of tau. It has been suggested that phosphorylation of tau at S199, S202, T205, T212, S235, S262 and S404 is required for the sequestration of normal tau, whereas further phosphorylation at positions T231 and S396 is needed for self-assembly. Phosphorylation at T181 and T217 are also suggested to be involved in self-assembly[29]. Whether other serine or threonine residues that are usually phosphorylated in ADP-tau are also phosphorylated by QA treatment remains to be investigated.

The alternative splicing of the tau gene produces six isoforms of tau in adult brain. The six isoforms of tau differ from each other by the presence or absence of one or two inserts in the N-terminal part and by the presence of either three or four repeats in the C-terminal region. Whereas all six isoforms are seen in adult brain, only the shortest isoform of tau is expressed in foetal brain [20]. Although we tested the effect of QA on foetal tau phosphorylation, our results can be extrapolated to adult tau for two reasons. Firstly, about one third of total tau in adult brain is the shortest (foetal) isoform (0N/3R)[20]; and secondly, most of the phosphorylation sites in PHF-tau are also phosphorylated in foetal brain tau[30], [31], [32]. In the AD brain, tau can be hyperphosphorylated by either an increase in the activities of one or more serine/threonine kinases or reduced activity of a phosphoseryl/phosphothreonyl protein phosphatase(s). The role of the kinases in tau hyperphosphorylation has been well studied but increase in activity of any one of these kinases has never been reproducibly shown in AD brain. Phosphoseryl/phosphothreonyl protein phosphatases are among the major regulators of the phosphorylation of tau[33], [34], [35]. Impairment of protein phosphatases not only directly affects the dephosphorylation of tau but may also up-regulate the activity of some protein kinases, which are phosphorylation dependent[35], [36], [37] [38] [39] [40].

The major serine/threonine phosphatases in the brain are PP1, PP2A and PP2B[41], [42], [43], [44]. Although quantitatively PP2B is the most abundantly expressed in the brain, it represents, due to specific activity, a small proportion of total brain phosphatases activity; PP1 and PP2A together account for over 90% of the total mammalian brain protein phosphatase activity, and any alteration in the activity of these two phosphatases may significantly affect the phosphorylation state of tau. A decrease of about 20% in the activities of PP1 and PP2A has been reported in AD brain[45], [46]. The observed increase in QA-induced phosphorylation of tau could be due to a decrease in the expression or activity of the major tau phosphatases. We measured total and OA-inhibited (PP2A) phosphatase activity in QA-treated neuronal lysate. We also measured the activity of immunoprecipitated PP2A from QA-treated neuronal lysate. We observed a biphasic effect on total phosphatase activity, using pNPP as substrate. The activity was increased at 100 nM QA but higher concentrations decreased the activity in a dose-dependent manner. However, PP2A activity (measured indirectly as OA-inhibited phosphatase activity and directly by immunoprecipitating PP2A and measuring its activity) was markedly decreased in a dose dependent manner.

We measured the expression level of PP1, PP2A, PP2B and PP5. QA treatment decreased the expression of PP1, PP2A and PP5 in a dose dependent manner. These data suggest that the observed reduction in phosphatase activity and subsequent tau phosphorylation is due to a decrease in the expression of the major tau phosphatases. The expression of PP2B, on the other hand, was markedly increased. The observed increase in total phosphatase activity with lower concentrations of QA (100–200 nM) could be due to an increase in PP2B activity. Liu et al. [44] have reported that PP2A, PP1, PP5 and PP2B account for ∼71%, ∼11%, ∼10% and ∼7%, respectively, of the total tau phosphatase activity of human brain. Increased expression of PP2B could be a compensatory mechanism for the substantial decrease in PP2A and PP1 activities. Our results are in accordance with the published literature reporting the increased expression of PP2B in AD brain, which is characterized by tau hyperphosphorylation. Up-regulation of PP2B expression in the pyramidal neurons of hippocampus in AD brain has been previously reported[47]. An increase in the catalytic cleavage and subsequent increase in the activity of PP2B in AD brain has also been reported[44].

Since QA is an NMDA receptor agonist, we investigated whether other agonists had a similar effect on tau phosphorylation. Both glutamate and NMDA increased tau phosphorylation at sites similar to QA. An increase in tau phosphorylation by glutamate has been reported previously[48]. We also studied the effects of various NMDA receptor antagonists on QA-induced tau phosphorylation. Of the antagonists studied, only memantine almost completely reversed QA-induced tau phosphorylation. MK-801 inhibited tau phosphorylation at AT-180 site but not at AT8 site, whereas, AP-5 had no effect at either site. Inhibition of okadaic acid-induced tau phosphorylation to the control level by memantine but not by other antagonists (5,7-dichlorokynurenic acid (DK) or with D(-)-2-amino-5-phosphonopentanoic acid (AP) in organotypic cultures of rat hippocampal slices has been reported by Li et al.^52^ and this observation is consistent with our findings. In this study memantine also reversed the OA-induced decrease in PP-2A activity, increase in calcium, calmodulin-dependent protein kinase II (CaMKII) and cyclin AMP-dependent PKA activities, the decrease and aggregation of MAP-2 and the phosphorylation and aggregation of neurofilament heavy/medium (NF-H/M) subunits and inhibited the neurodegeneration associated with these changes. Memantine is a low to moderate affinity non-competitive NMDA receptor channel blocker that leads to functional improvement and reduces care dependence in moderate to severe AD patients[49], [50]. The NMDA receptor has been reported to be associated with PP-2A and stimulation of this receptor can lead to the dissociation of PP-2A from the complex and the reduction of PP-2A activity in cultured cells[51]. It has been suggested that, in addition to its activity as an NMDA receptor antagonist, the effects of memantine might involve modulation of PP-2A signalling, a property not shared by the other NMDA receptor antagonists[48].

**Figure 10:**
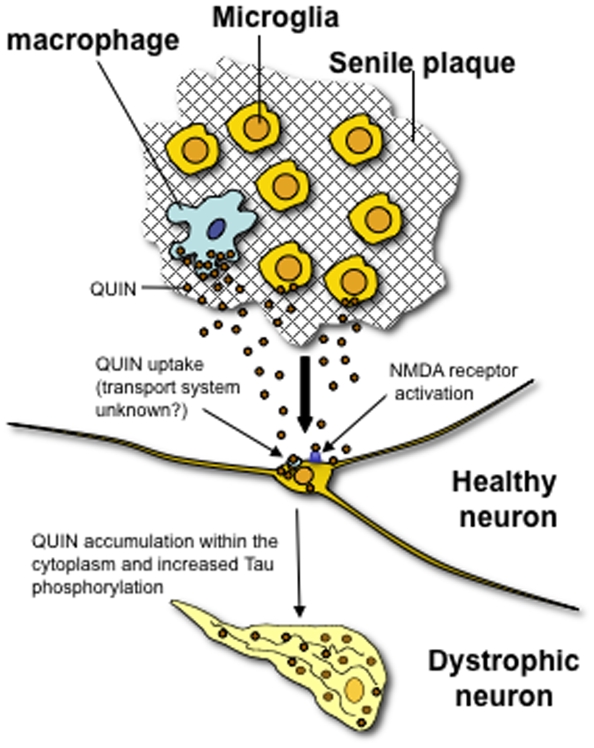
hypothetical model of QA effects on Tau phosphorylation in primary human neurons.

Finally, we used PCR array to determine which of the genes associated with tau phosphorylation may be switched on by QA. We found that QA at the sub-physiological concentrations found in amyloid plaques[26] was able to activate 10 genes in primary human neurons (Fig. 10; table 2). Six of these genes e.g. CDK5, MYC, WNT1, IL1α, iNOS and BRCA1 are associated with intracellular pathways known to play a role in tau phosphorylation (Table 2). The other genes WISP1, LEP, C/EBPB and CSF2 are known to be associated with neuroprotective mechanisms and are logically activated in response to QA.

In conclusion, we showed in this study that QA over-production by activated microglia/macrophages and consequent accumulation in AD brain is likely to be involved in two mechanisms 1) promotion of tau pathology by inducing tau hyperphosphorylation and 2) direct excitotoxicity (Figure 10). We propose that a decrease in major tau phosphatases (PP2A, PP1 and PP5) is a mechanism for this increased in tau phosphorylation in neurons exposed to QA at pathophysiological concentrations. This study has identified a new mechanism involved in AD neurodegeneration and may open a new therapeutic avenue for treatment of AD.

## Supporting Information

Figure S1 QA uptake by human neurons in vitro. These data demonstrate that QUIN is taken up by human primary neurons in vitro.
(0.29 MB PDF)

Figure S2 Determination of QPRTase activity in human neuron. We developed a new method to quantify QPRTase activity and we found that neuronal QPRTase activity starts to be saturated at concentrations above 300 nM.
(0.15 MB PDF)

Figure S3 Intracellular co-localization of neurofibrillary tangle and QA in a human neuron. Serial optical sections were taken through the neuron at 2 µm intervals. QA (red; Alexa 647) and tau (AT8; green; Alexa 594).
(0.60 MB PDF)
