# Expression of Concern: The Excitotoxin Quinolinic Acid Induces Tau Phosphorylation in Human Neurons

**DOI:** 10.1371/journal.pone.0314139

**Published:** 2024-11-14

**Authors:** The PLOS ONE Editors

After this article [1] was published, concerns were raised about results presented in Figs 4 and 8. Specifically:

- The Tau panels in Fig 4A–4C appear similar to each other.
- There appears to be a vertical discontinuity between lanes 4 and 5 in the AT8 blot in Fig 8A.
- There appear to be vertical discontinuities between lanes 1 and 2 in the Tau and AT180 blots in Fig 8B.

The authors did not respond to queries about the experiments in Figs 4 and 8.

In light of the unresolved concerns in Figs 4 and 8, the *PLOS ONE* Editors issue this Expression of Concern.

## References

[1] Rahman A, Ting K, Cullen KM, Braidy N, Brew BJ, Guillemin GJ (2009) The Excitotoxin Quinolinic Acid Induces Tau Phosphorylation in Human Neurons. PLoS ONE 4(7): e6344. 10.1371/journal.pone.0006344 19623258 PMC2709912

