# Supplementary material for: The Excitotoxin Quinolinic Acid Induces Tau Phosphorylation in Human Neurons
Source: PLoS One. 2009 Jul 22;4(7):e6344. doi: 10.1371/journal.pone.0006344 (PMC2709912; doi:10.1371/journal.pone.0006344)
Supplement: Figure S1 — QA uptake by human neurons in vitro. These data demonstrate that QUIN is taken up by human primary neurons in vitro. (0.29 MB PDF) [file pone.0006344.s001.pdf]

SUPPLEMENTARY DATA 1

QUIN is taken up by human primary neurons *in vitro*.

Methods

Human foetal neurons were grown in slide flasks for 7 days. QA (0, 5, 15, 30, 60 and 90 min) was added to cells, followed by washes with PBS. Cells were then fixed with acetone/methanol (vol/vol) at -20ºC for 10 min. QA was detected using immunocytochemistry with a QUIN mAb (Millipore). We used confocal microscopy to distinguish QA internalisation versus association with the cell membrane (unknown receptors/transporters).

Results

At t=0, no QA immunolabelling was detected. The staining progressively appeared after 30 min and was mostly localized extracellularly (cell membrane and neurites). After 1hour, QA labelling became more intense and localized both extra- and intracellularly. Finally, after 90 min, QA staining appeared more intense in the cytoplasm and vacuoles containing QA were observed in dystrophic neurons.

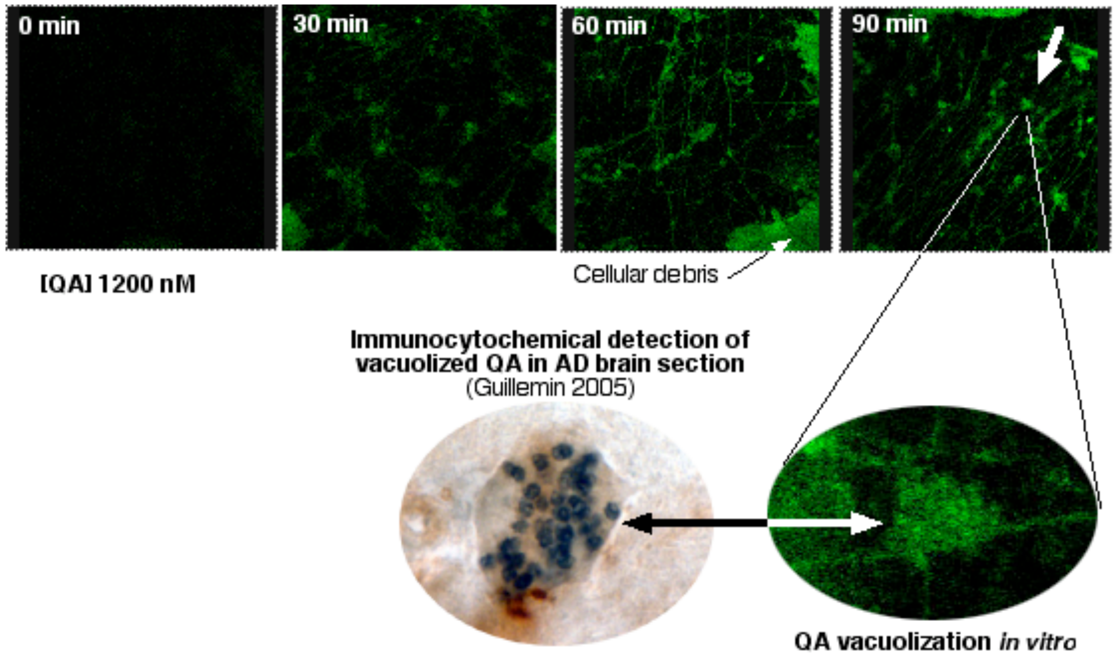

Discussion

These results demonstrated the neuronal ability to uptake QA. The lack of staining at t=0 confirmed our previous data showing that human primary neurons are unable to produce QA [1, 2]. We previously demonstrated that within the brain, only infiltrating macrophages and activated microglia produce QA [1, 3-5].

It is important to note that mechanisms of QUIN uptake by any cell type are unknown. There are few potential candidates for this function. Among them the two newly described excitatory amino acid transporters GLAST1b and exon 9 skipping EAAT3 expressed specifically by neurons subject to abnormal excitatory drive[6].

There are also other potential mechanisms including organic anion transporter 1 (OAT1)[7], clathrin-dependent endocytosis, and NMDA receptor. Of note, QUIN is known to activate NMDAR[8], suggesting that NMDAR-mediated uptake is a feasible candidate to investigate.

We have shown that QUIN accumulates in the AD brain, particularly in dystrophic neurons[9]. We predict that this likely represents the neuronal uptake of extracellular QA and may lead to 1) tau hyperphosphorylation and 2) trigger a cytotoxic cascade within neurons ultimately leading to neurodegeneration.

References

1. Guillemin, G.J., Smythe, G., Takikawa, O. & Brew, B.J. Expression of indoleamine 2,3-dioxygenase and production of quinolinic acid by human microglia, astrocytes, and neurons. *Glia* **49**, 15-23 (2005).
2. Guillemin, G.J. *et al.* Characterization of the Kynurenine Pathway in Human Neurons. *J Neurosci* **27**, 12884-12892 (2007).
3. Guillemin, G.J., Smith, D.G., Smythe, G.A., Armati, P.J. & Brew, B.J. Expression of the kynurenine pathway enzymes in human microglia and macrophages. *Adv Exp Med Biol* **527**, 105-112 (2003).
4. Guillemin, G.J., Smythe, G.A., Veas, L.A., Takikawa, O. & Brew, B.J. A beta 1-42 induces production of quinolinic acid by human macrophages and microglia. *Neuroreport* **14**, 2311-2315 (2003).
5. Guillemin, G.J. & Brew, B.J. Microglia, macrophages, perivascular macrophages, and pericytes: a review of function and identification. *J Leukoc Biol* **75**, 388-397 (2004).
6. Sullivan, S.M., Macnab, L.T., Bjorkman, S.T., Colditz, P.B. & Pow, D.V. GLAST1b, the exon-9 skipping form of the glutamate-aspartate transporter EAAT1 is a sensitive marker of neuronal dysfunction in the hypoxic brain. *Neuroscience* **149**, 434-445 (2007).
7. Bahn, A. *et al.* Murine renal organic anion transporters mOAT1 and mOAT3 facilitate the transport of neuroactive tryptophan metabolites. *Am J Physiol Cell Physiol* **289**, C1075-1084 (2005).
8. Guillemin, G.J., Kerr, S.J. & Brew, B.J. Involvement of quinolinic acid in AIDS dementia complex. *Neurotox Res* **7**, 103-123 (2005).
9. Guillemin, G.J., Brew, B.J., Noonan, C.E., Takikawa, O. & Cullen, K.M. Indoleamine 2,3 dioxygenase and quinolinic acid immunoreactivity in Alzheimer's disease hippocampus. *Neuropathol Appl Neurobiol* **31**, 395-404 (2005).
