# Supplementary material for: The Excitotoxin Quinolinic Acid Induces Tau Phosphorylation in Human Neurons
Source: PLoS One. 2009 Jul 22;4(7):e6344. doi: 10.1371/journal.pone.0006344 (PMC2709912; doi:10.1371/journal.pone.0006344)
Supplement: Figure S2 — Determination of QPRTase activity in human neuron. We developed a new method to quantify QPRTase activity and we found that neuronal QPRTase activity starts to be saturated at concentrations above 300 nM. (0.15 MB PDF) [file pone.0006344.s002.pdf]

SUPPLEMENTARY DATA 2

QPRTase Activity Assay

QPRTase activity in human foetal neurons was determined by measuring the formation of nicotinic acid mononucleotide (NAMN) using a continuous UV spectrophotometric assay as previously described by Liu et al (2007). Briefly, human foetal neurons were washed twice and then resuspended in 50 mM K_2HPO_4/KH_2PO_4 solution containing 6 mM $MgCl_2$ (pH 7.2), and sonicated for 10 seconds. Afterwards, the cell homogenate was centrifuged for 10 minutes (1000 g, 37°C). The reaction mixture contained 50 mM K_2HPO_4/KH_2PO_4 solution containing 6 mM $MgCl_2$ (pH 7.2), 0.025 mM PRPP, and varying concentrations of QUIN (0-1100 nM) in a total volume of 1 mL. After adding 20 µg of endogenous neuronal QPRTase, the mixtures were incubated at 37°C for 30 minutes. The increase in absorbance for 30 minutes was then recorded continuously at 266 nm using the Cary 50BIO UV spectrophotometer (Varian, Sydney). Change in absorbance per minute was calculated from the linear progress curve and the amount of NAMN produced per minute (µM/min) was determined using an NAMN standard curve. Each data point was measured in quadruplicate. Kinetic parameters were calculated using *Graphpad Prism 3* program using non-linear regression analysis of the quadruplicate experimental data.

Results

Figure 1. Effect of QUIN concentration on the rate of formation of NAMN by human neuronal QPRTase. (A) Michelson-Menton plot for QUIN at a fixed PRPP concentration. (B) Lineweaver-Burk plot for QUIN using a fixed PRPP concentration. (A)

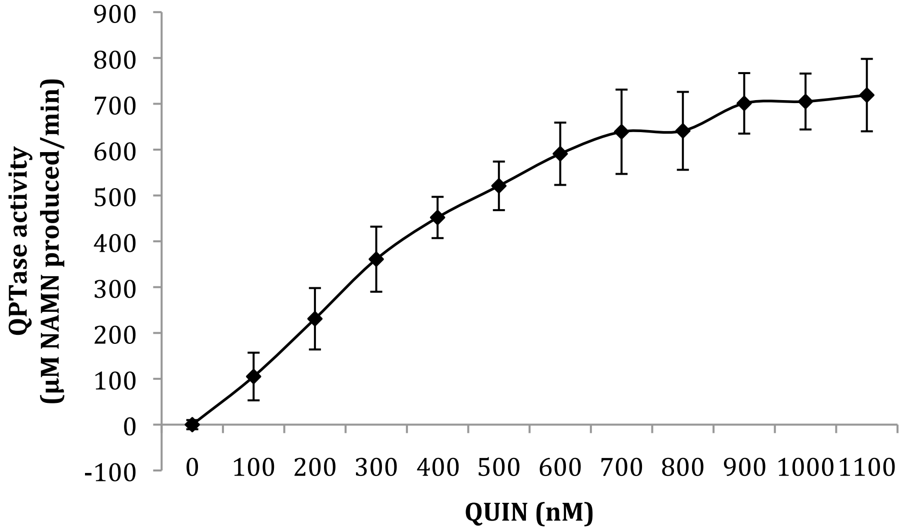

(B)

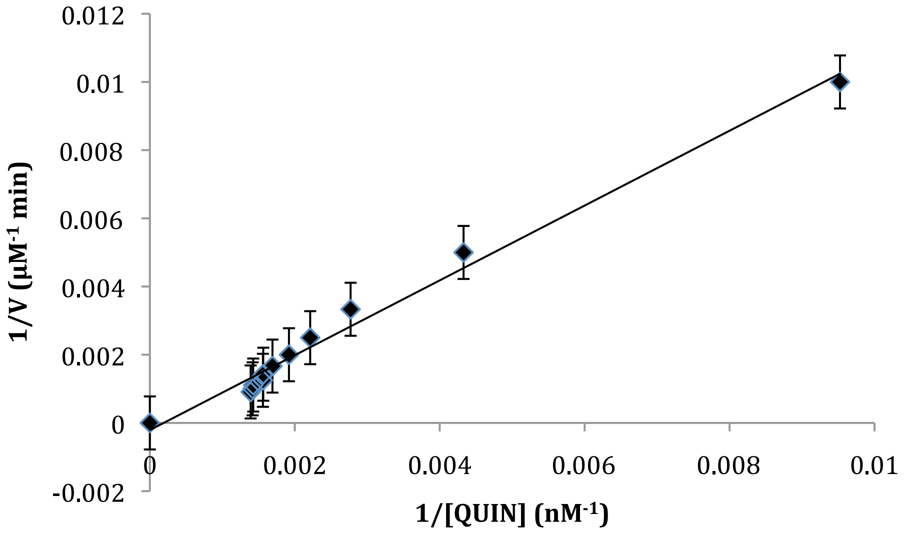

QPRTase activity in human primary foetal neurons treated with QUIN (0-1100 nM)

QPRTase activity was determined using a continuous UV-spectrophotometric assay. Kinetic parameters for QUIN were determined using 0.025 mM PRPP and found to be Km = 260.3 (± 24.1) nM and Vmax = 719.5 (± 30.6) μM/min. The enzyme reaction is first-order at concentrations below 300 nM, but at higher concentrations, the enzyme reaction exhibits zero-order kinetics with respect to the QUIN concentration. This suggests that neuronal QPRTase activity starts to be saturated at concentrations above 300 nM.

Discussion

Elevations of QUIN in the CNS have been associated with the pathogenesis of several neurodegenerative diseases including AD [1, 2]. Given the significance of QUIN as both a precursor for NAD^+ synthesis and a putative neurotoxin, the neurotoxic effects of QUIN may be attributed to the kinetics of QUIN production and degradation. The hypothesis is further strengthened by the fact that no specific removal or uptake of QUIN has been identified in the CNS [3]. QUIN is produced by 3-hydroxyanthranilic acid oxygenase (3-HAAO) (EC 1.12.11.6) which catalyses the oxidative cleavage reaction of 3-hydroxyanthranilic acid (3-HA) to α-amino-β-carboxymuconate-ε-seminal-dehyde (ACMS). ACMS undergoes cyclisation to form QUIN [4]. The activity of 3-HAAO is elevated in several neurological disorders including Huntington's disease [5] and epilepsy [6].

QUIN levels are reduced by quinolinate phosphoribosyltransferase (QPRTase) (EC 2.4.2.19). QPRTase converts QUIN to nicotinic acid mononucleotide (NaMN). NaMN is then adenylated by nicotinamide mononucleotide adenyltransferases (NMNAT1-3) to form nicotinic acid adenine dinucleotide ($NaAD^+$), which is subsequently converted to NAD^+ by glutamine-dependent NAD^+ synthetase [4]. Our present data implies that QPRTase activity starts to be saturated in the presence of high extracellular levels of QUIN (> 300 nM). The normal physiological concentration of QUIN in the brain and cerebral spinal fluid (CSF) is usually less than 100 nM [3], and higher levels can induce oligodendrocyte, neuronal and astrocytic apoptosis [7-9]. In addition, intrastriatal injections of QUIN at 350 nM in rats for 5 weeks has been shown to induce detrimental changes in the neuronal cytoskeleton

including the formation of dendritic varicosities and disruption to microtubule morphology.
We have previously shown that astrocytes can only degrade up to half the concentration of QUIN (~1 μM) added in the cultures [10]. If the present catabolic factor is extrapolated to the amount of QUIN produced by activated macrophages after IFN-γ stimulation for 48 hours, the ability of astroglial and neuronal QPRTase to catabolise QUIN to NAD^+ is significantly reduced [10]. Unlike glutamate, there appears to be no effective mechanism to remove excess extracellular QUIN. Therefore, minor changes in QUIN levels may have a deleterious effect on neuronal cells.

References:

1. Guillemin, G.J. & Brew, B.J. Implications of the kynurenine pathway and quinolinic acid in Alzheimer's disease. *Redox Rep* 7, 199-206. (2002).
2. Stone, T.W. Endogenous neurotoxins from tryptophan. *Toxicon* 39, 61-73 (2001).
3. Stone, T.W., Forrest, C.M., Mackay, G.M., Stoy, N. & Darlington, L.G. Tryptophan, adenosine, neurodegeneration and neuroprotection. *Metab Brain Dis* (2007).
4. Liu, H. *et al.* Structural and Kinetic Characterization of Quinolinate Phosphoribosyltransferase (hQPRTase) from Homo sapiens. *J Mol Biol* (2007).
5. Speciale, C., Okuno, E. & Schwarcz, R. Increased quinolinic acid metabolism following neuronal degeneration in the rat hippocampus. *Brain Res* 436, 18-24 (1987).
6. Feldblum, S. *et al.* Quinolinic-phosphoribosyl transferase activity is decreased in epileptic human brain tissue. *Epilepsia* 29, 523-529 (1988).
7. Kelly, W.J. & Burke, R.E. Apoptotic neuron death in rat substantia nigra induced by striatal excitotoxic injury is developmentally dependent. *Neurosci Lett* 220, 85-88 (1996).
8. Cammer, W. Apoptosis of oligodendrocytes in secondary cultures from neonatal rat brains. *Neurosci Lett* 327, 123-127 (2002).
9. Guillemin, G.J., Wang, L. & Brew, B.J. Quinolinic acid selectively induces apoptosis of human astocytes: Potential role in AIDS dementia complex. *J Neuroinflammation* 2, 1-6 (2005).
10. Guillemin, G.J. *et al.* Kynurenine pathway metabolism in human astrocytes: a paradox for neuronal protection. *J Neurochem* 78, 1-13 (2001).
