# Supplementary material for: The Excitotoxin Quinolinic Acid Induces Tau Phosphorylation in Human Neurons
Source: PLoS One. 2009 Jul 22;4(7):e6344. doi: 10.1371/journal.pone.0006344 (PMC2709912; doi:10.1371/journal.pone.0006344)

Supplementary figure 3

QA (red; Alexa 647) and tau (AT8; green; Alexa 594) colocalisation in a neuron containing neurofibrillary tangle. and QA. Optical sections were taken through the neuron at 2 µm intervals.

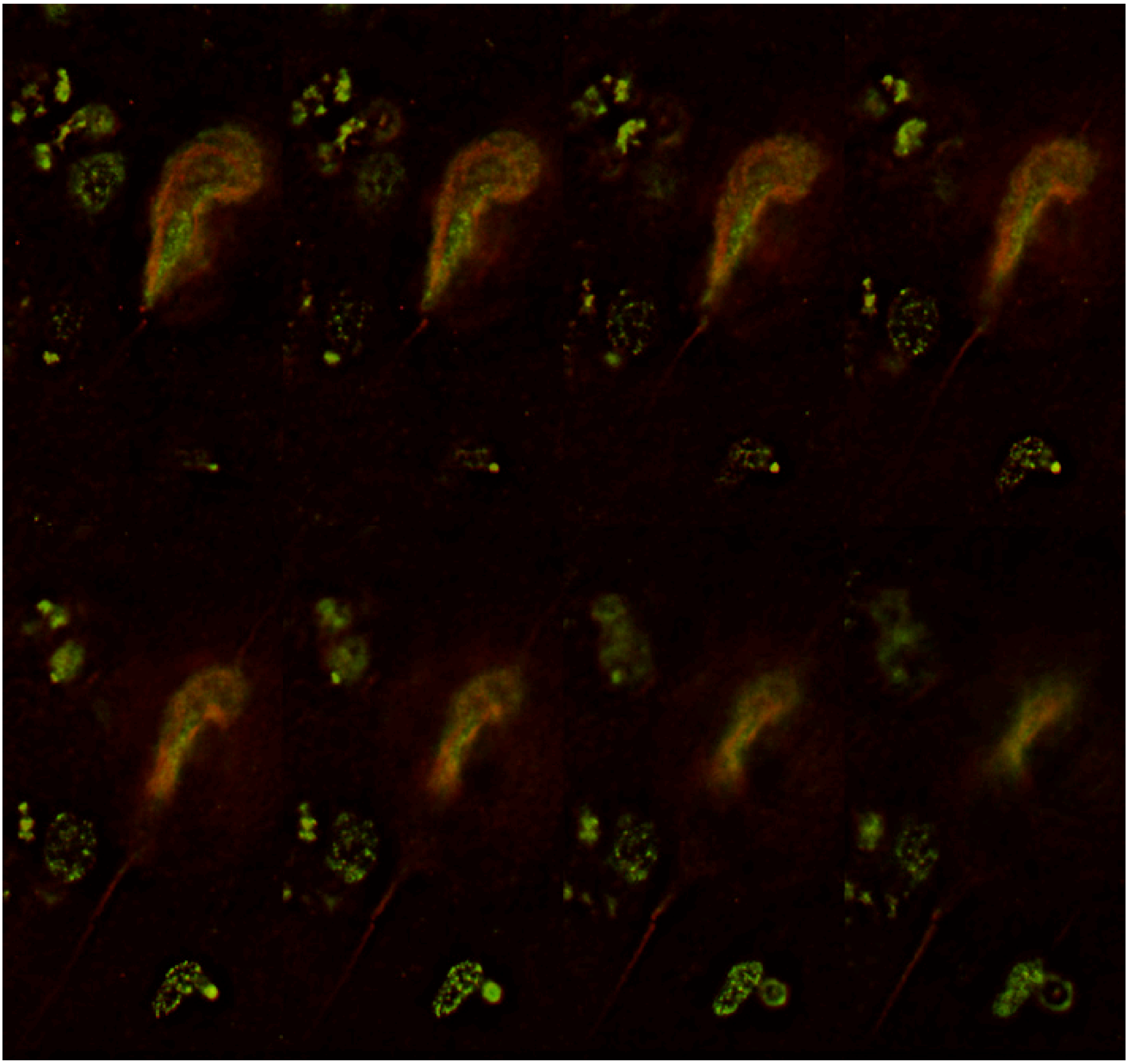

Supplement: Figure S3 — Intracellular co-localization of neurofibrillary tangle and QA in a human neuron. Serial optical sections were taken through the neuron at 2 µm intervals. QA (red; Alexa 647) and tau (AT8; green; Alexa 594). (0.60 MB PDF) [file pone.0006344.s003.pdf]
